# The survival penalty: Age-based inequities in hypertension and diabetes management in African health systems (1990–2022) – a secondary quantitative analysis

**DOI:** 10.1371/journal.pgph.0006306

**Published:** 2026-07-23

**Authors:** Lawrence Ejike Ugwu, Bruno Basil, Uzoamaka Francisca Ugwoke, Effiness Harawa Kamanga, Ngozi Ijeoma Okoro

**Affiliations:** 1 Centre for Applied Psychology and Public Health Research in Africa (CAPPHRA), Enugu, Nigeria; 2 International Institute for Pathology and Forensic Science Research, David Umahi Federal University of Health Sciences, Uburu, Nigeria; 3 Department of Psychology, Enugu State University of Science and Technology, Enugu, Nigeria; 4 Self-directed Learning, Faculty of Education, North-West University, Mafikeng, South Africa; 5 Department of Chemical Pathology, Enugu State University of Science and Technology, Enugu, Nigeria; Directorate of Factories, INDIA

## Abstract

Africa is experiencing rapid population ageing, yet many health systems remain oriented towards acute and infectious disease care. Whether population-level indicators of hypertension and diabetes management remain comparable across older age groups is unclear. We assessed age-associated differences in chronic disease care cascade indicators across African Union member states using age-stratified estimates from the NCD Risk Factor Collaboration (NCD-RisC). We analysed country-year estimates for hypertension (1990–2019) and diabetes (1990–2022), comparing a working-age reference group (50–54 years), a young-old group (65–69 years), and the oldest available age groups (75–79 years for hypertension and ≥85 years for diabetes). Outcomes included diabetes treatment coverage, hypertension diagnosis, treatment and control, and the prevalence of severe untreated hypertension (stage 2; BP > 160/100 mmHg). The analysis included NCD-RisC estimates derived from 2,309 population-based studies involving 245 million participants. Hypertension prevalence was higher in adults aged 75–79 years than those aged 50–54 years (64·4% vs 42·6%; relative risk 1·51). Although diagnosis was more frequent among older adults (53·3% vs 42·6%), hypertension control remained lower (11·6% vs 16·9%). Diabetes treatment coverage declined after age 70, falling from 32·9% in adults aged 65–69 years to 23·1% in those aged ≥85 years (difference 9·8 percentage points; 95% UI 5·9–13·7). Severe untreated hypertension was more common in adults aged 75–79 years than in those aged 50–54 years (29·8% vs 26·6%), with the highest prevalence in Central and West Africa. Across Africa, diabetes treatment coverage and hypertension control declined with advancing age despite higher diagnosis rates, while severe untreated hypertension remained common among older adults. These findings support incorporating age-specific chronic disease cascade indicators into Universal Health Coverage monitoring and strengthening health systems to improve cardiovascular risk management in ageing populations.

## Research in context

### Evidence before this study

We searched PubMed and Google Scholar for studies published between Jan 1, 1990, and Dec 31, 2024, using terms including “Africa”, “hypertension”, “diabetes”, “older adults”, “elderly”, “health system performance”, “cascade of care”, and “age inequities”. While there is robust evidence documenting the rising burden of non-communicable diseases (NCDs) in Africa and the demographic shift towards an ageing population, most health system analyses treat “adults” as a homogeneous group or focus primarily on prevalence. Few studies have explicitly compared care cascade indicators in the “oldest-old” (e.g.,  ≥≥ 75 or ≥80 years) with younger adult cohorts across multiple African countries, and evidence on factors contributing to observed age gradients remains limited and fragmented.

### Added value of this study

This is, to our knowledge, the first continental analysis to describe age-stratified inequities in NCD management across all 54 African Union member states using harmonised NCD-RisC estimates for hypertension (1990–2019) and diabetes (1990–2022). We quantify age-associated differences in diabetes treatment coverage after age 70 and examine hypertension cascade indicators alongside the burden of severe untreated hypertension (BP  >> 160/100 mmHg) as a public-health risk marker. We also describe geographic and sex-stratified heterogeneity in these patterns and identify countries with comparatively smaller age gradients as potential case studies for further investigation.

### Implications of all the available evidence

The evidence indicates that Universal Health Coverage (UHC) expansion may be associated with uneven patterns across age groups if age-specific retention and treatment intensity are not monitored. Because treatment targets and clinical decision-making may differ in very old or frail adults, age gradients should be interpreted cautiously; however, high levels of severe untreated hypertension remain a clear signal of avoidable risk. Policy and programmatic responses should include age-responsive monitoring of cascade indicators, strengthened continuity of chronic care, and safeguards to ensure older adults are not disproportionately disadvantaged in access to care.

## Introduction

Africa is entering rapid population ageing, yet its health systems were built for acute, maternal, and infectious disease care [1,2]. Whether these systems can deliver long-term, equitable chronic care to older adults is unclear. As life expectancy increases across the continent, the burden of non-communicable diseases (NCDs) is shifting toward later life [2,3]. However, current Universal Health Coverage (UHC) frameworks often prioritise coverage expansion and enrolment metrics [4], and it remains uncertain whether gains in detection translate into sustained treatment and control for the oldest age groups [5]. We use the term “survival penalty” as a descriptive construct for age-associated shortfalls in cascade performance at advanced ages, without implying a specific underlying mechanism.

Emerging signals raise the possibility that chronic care cascades may show steeper age-related declines in some African settings [6–8]. We hypothesised that diabetes treatment coverage may decline after age 70 (“post-70 drop-off”), and that hypertension management may show a substantial diagnosis–control gap in older adults [9,10]. Because the present study uses aggregated, modelled estimates, we interpret observed patterns as descriptive age gradients and do not infer causality or underlying system intent.

Herein, we evaluate national health system performance across 54 African Union member states to quantify age-based differences in NCD management. Using aggregated, age-stratified estimates from the NCD Risk Factor Collaboration (NCD-RisC) covering hypertension (1990–2019) and diabetes (1990–2022), we describe how cascade indicators vary by age, sex, and subregion, and we identify the points in the cascade where older-age differences are most pronounced.

## Methods

### Ethics statement

This research involves a secondary quantitative analysis of publicly available, aggregated, and anonymized population health estimates. As the study utilized data that contained no individual-level identifiers and was accessed via the NCD Risk Factor Collaboration (NCD-RisC) public repository, it was exempt from institutional review board (IRB) approval. Participant recruitment, ethical oversight, and the acquisition of formal consent were conducted by the primary investigators of the original population-based surveys synthesized by NCD-RisC. Formal consent for this specific secondary analysis was not obtained because the data were fully anonymized and aggregated at the national, age, and sex levels prior to our access and analysis.

### Study design

This study is a secondary, ecological analysis of aggregated, age-stratified population health estimates for the 54 African Union member states. We utilised posterior mean estimates from the NCD Risk Factor Collaboration (NCD-RisC), accessed between 12th and 25th October 2025, to describe age patterns in hypertension and diabetes care cascade indicators. Because inputs are modelled and aggregated, analyses describe population-level gradients and do not support causal attribution about individual clinical decisions or intent. The study follows the Guidelines for Accurate and Transparent Health Estimates Reporting (GATHER) and relevant STROBE reporting items for observational analyses.

### Data sources and handling

Two specific datasets were analysed:

1. Hypertension (1990–2019): Estimates of prevalence, diagnosis, treatment, and control rates were derived from 1,201 population-based studies involving 104 million participants.
2. Diabetes (1990–2022): Estimates of prevalence and treatment coverage were derived from 1,108 comparable national surveys involving 141 million participants.

The NCD-RisC estimates are generated using a Bayesian hierarchical model that incorporates nonlinear time trends and age patterns to account for data scarcity in select nations; the underlying model inputs incorporate study-level adjustments and, where applicable, survey sampling weights as described in NCD-RisC methodological documentation. We used the country-year-age group estimates without further imputation.

### Procedures and definitions

To evaluate age-associated gradients in care cascade indicators, we stratified the population into three functional age categories based on available NCD-RisC age strata:

1. The Workforce (Reference Group): Adults aged 50–54 years, selected as a stable late-career working-age reference group for cross-country comparison.
2. The “Young-Old”: Adults aged 65–69 years, representing the transition to retirement.
3. The “Oldest-Old”: Adults aged 75–79 years for hypertension (the oldest 5-year band available in the hypertension cascade estimates) and ≥85 years for diabetes (the oldest open-ended stratum available in the diabetes treatment estimates).

### Outcomes

Primary outcomes were age-related differences in chronic care cascade indicators (descriptive):

1. Diabetes treatment coverage drop-off: Diabetes treatment coverage in 2022, summarised as the percentage-point difference between the “Young-Old” (65–69 years) and the “Oldest-Old” (≥85 years). Larger positive differences indicate larger age-related gaps in treatment coverage.
2. Hypertension cascade gaps and severe untreated burden: Using hypertension estimates in 2019, we describe (i) diagnosis, treatment, and control levels; (ii) the diagnosis–control gap as a summary of downstream management shortfalls; and (iii) the prevalence of severe untreated (stage 2) hypertension (systolic BP > 160 mmHg or diastolic BP > 100 mmHg) as a public-health risk marker. These indicators do not isolate specific failure points (e.g., initiation vs adherence vs intensification) and should not be interpreted as direct measures of clinician behaviour. Control thresholds follow standard guideline definitions for hypertension management in adults [11].

### Sub-regional and gender analysis

Countries were stratified into five subregions (Central, East, North, Southern, and West Africa) according to African Union designations. Sex-stratified analyses were conducted to describe whether age gradients differed between men and women.

### Statistical analysis

The unit of analysis was the national health system (N = 54). We summarised country-level posterior mean estimates and computed percentage-point differences between age groups for each country (e.g., diabetes 65–69 minus ≥85; hypertension 75–79 minus 50–54 for severe untreated stage 2). We report descriptive statistics (means, ranges) and present graphical summaries and country rankings. As an additional sensitivity analysis and robustness check against cut-point sensitivity (to assess whether observed age-related patterns are sensitive to specific age thresholds rather than to infer causal effects), we visualise age-cohort profiles across all available age bands for key indicators (Fig 2A). We avoided conventional hypothesis testing because posterior uncertainty and cross-country dependence are not fully represented by point estimates alone.

**Fig 1:**
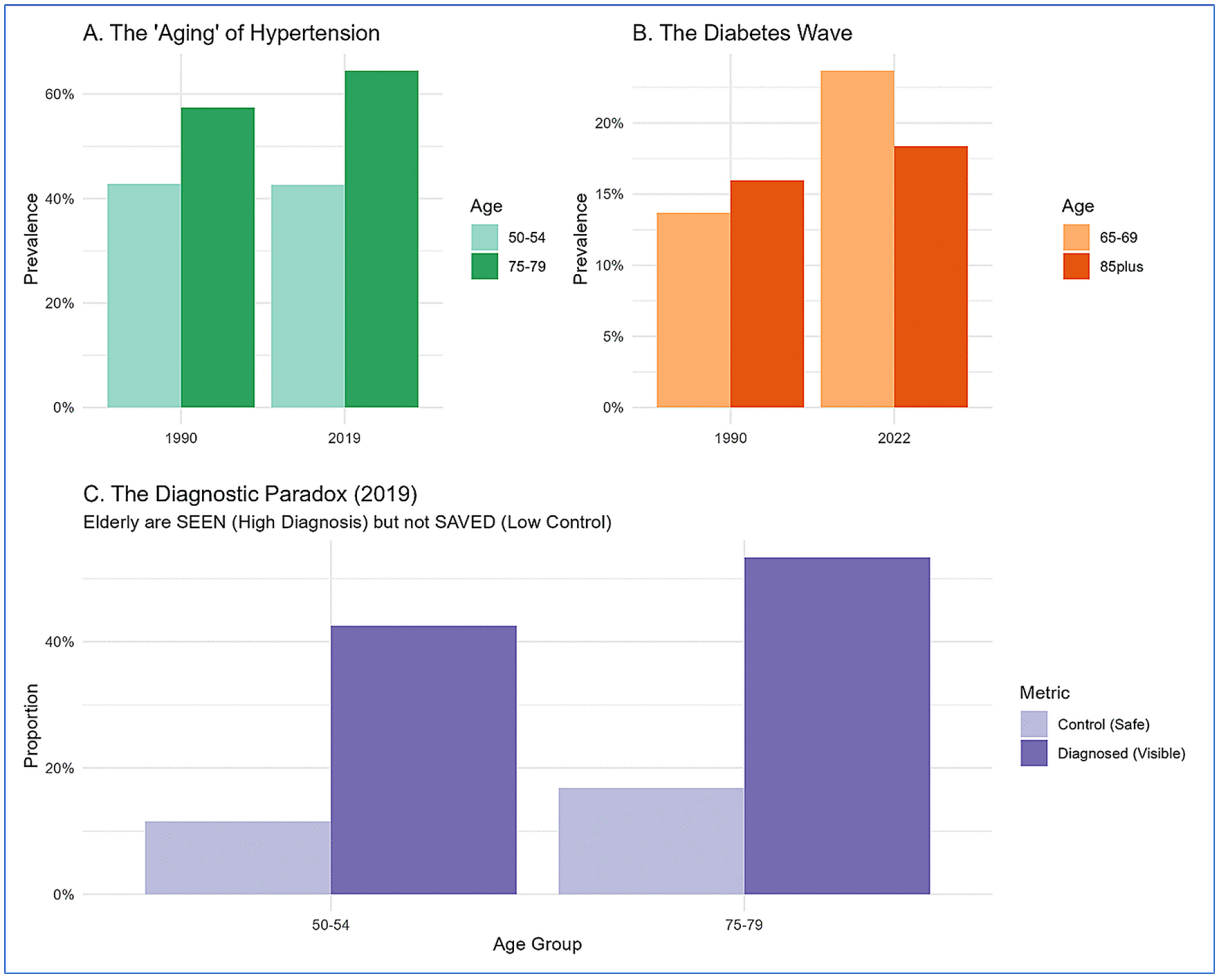
Descriptive epidemiology: ageing patterns in hypertension and diabetes and the diagnosis–control gap. (A) Hypertension prevalence in 1990 vs 2019, comparing 50–54 vs 75–79. (B) Diabetes prevalence in 1990 vs 2022, comparing 65–69 vs ≥ 85. (C) Diagnosis versus control for hypertension in 2019, comparing 50–54 vs 75–79.

Uncertainty propagation for derived differences: NCD-RisC provides posterior means and 95% uncertainty intervals (UIs) for each estimate. To propagate uncertainty into derived gaps (e.g., diabetes treatment coverage at ages 65–69 minus ≥85), we approximated the standard error (SE) from the published 95% UI as SE = (Upper UI − Lower UI) / (2 × 1·96). Assuming independence between age-stratified estimates within a given year (a conservative assumption because age-specific posteriors are likely positively correlated), the SE of an age-group difference was computed as SE diff = √(SE_A² + SE_B²), and the 95% UI for the gap as Gap mean ± 1·96 × SE_diff. Because uncertainty is widest for the oldest age bands at the country level, we additionally summarised interval-based differences at regional and continental levels. For these pooled analyses, we first derived a single country-level estimate for both sexes (using the NCD-RisC both-sex estimate where available; otherwise, averaging sex-specific estimates within country and pooling their SEs), then computed unweighted means across countries and pooled SEs for the mean as √(ΣSE_i²) / N. A gap was interpreted as supported by the modelled uncertainty when its 95% UI excluded zero. Analyses were performed using R (version 4.5.2).

## Results

### Study characteristics

Data were available for all 54 African Union member states. We analysed hypertension estimates (1990–2019) and diabetes estimates (1990–2022) to evaluate age-based inequities in health system performance.

### Hypertension burden, detection, and control

The burden of hypertension was concentrated in later life (Fig 1A). Mean prevalence was higher among older adults (75–79 years) compared with the working-age reference group (50–54 years) (64·4% vs 42·6%; relative risk 1·51) (Table 1). Diagnosis rates were higher in the 75–79 cohort (53·3%) than in the 50–54 cohort (42·6%). However, blood pressure control remained low in both groups (11·6% vs 16·9%), indicating a substantial difference between diagnosis and control levels (Fig 1C).

**Table 1:** Hypertension cascade indicators comparing adults aged 50–54 years with older adults aged 75–79 years.

Metric	Workforce (50–54 Years)	Geriatric (75–79 Years)	Relative Risk / Trend
Burden (Prevalence)	42.59%	64.4%	1.51 × higher prevalence in older adults.
Diagnosis/awareness (among those with hypertension)	42.61%	53.3%	Higher diagnosis in older adults (+10.7 percentage points).
Outcome (Control)	11.60%	16.9%	Low control in both groups.
Risk (Untreated Stage 2)	26.61%	29.8%	Higher severe untreated burden in older adults.

*Note: Prevalence and untreated stage 2 hypertension are population prevalences (% of all adults in the age band). Diagnosis/awareness, treatment, and control are cascade indicators expressed as proportions among individuals with hypertension in the same age band (as provided by NCD‑RisC). Because these quantities have different denominators, they should not be interpreted as directly comparable percentages.*

### Diabetes treatment coverage: Post-70 decline

**Fig 2:**
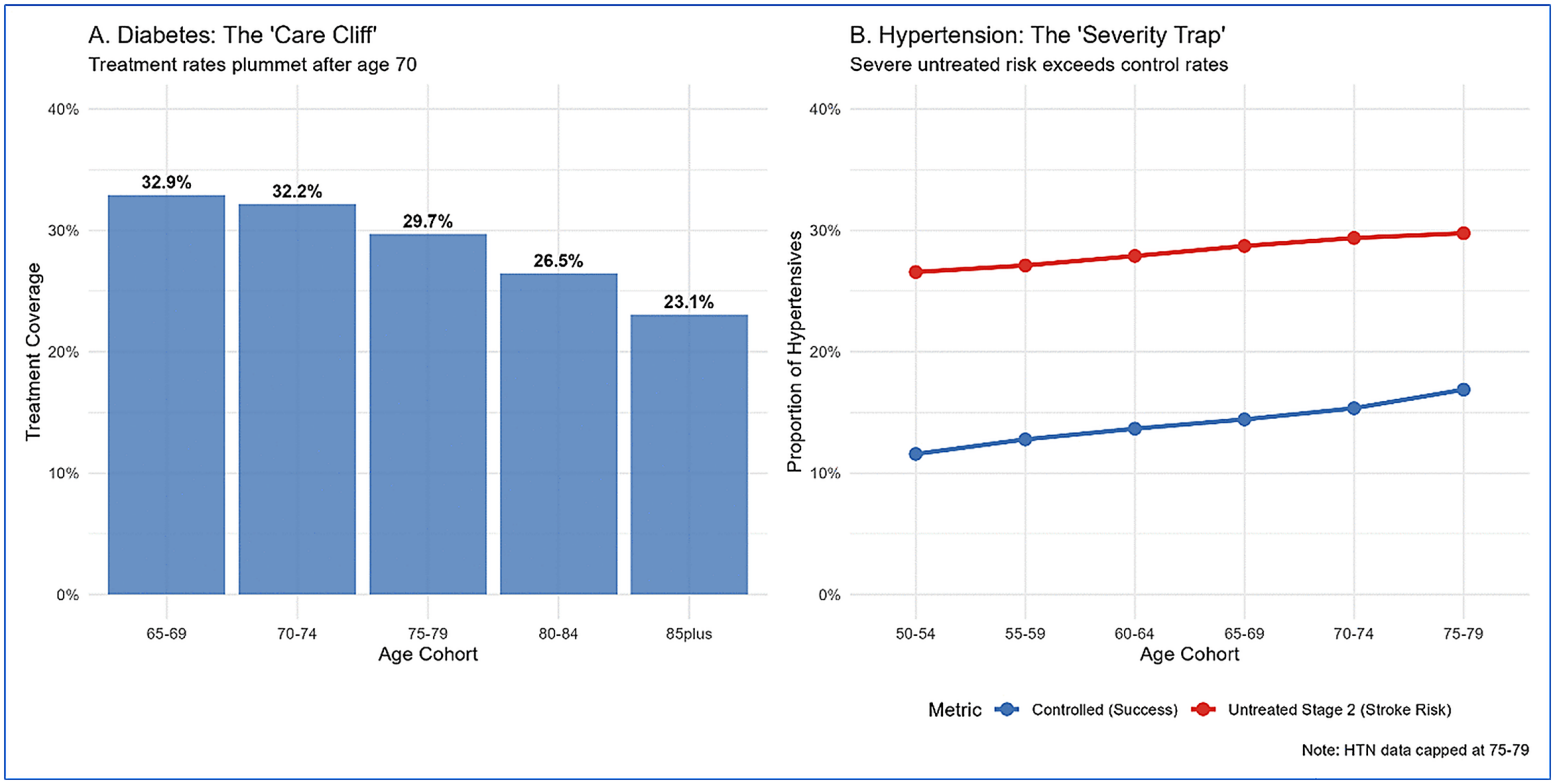
Age patterns in diabetes treatment coverage and severe untreated hypertension. (A) Diabetes treatment coverage by age cohort (2022), showing lower coverage at older ages. (B) Hypertension control and severe untreated stage 2 hypertension (2019) across age cohorts.

Diabetes prevalence was lower in the oldest-old (≥85 years) compared with the young-old (65–69 years) (Fig 1B). Treatment coverage nevertheless declined after age 70 (Fig 2A), from 32·9% in the 65–69 cohort to 23·1% in the ≥ 85 cohort (difference 9·8 percentage points). When propagating uncertainty from published 95% uncertainty intervals and aggregating across countries, the continental treatment coverage drop-off remained positive (9·8 percentage points; 95% UI 5·9 to 13·7).

### Inequities in severe clinical risk

To quantify severe clinical risk, we examined severe untreated hypertension (stage 2; BP > 160/100 mmHg). Among older adults (75–79 years), the prevalence of severe untreated hypertension was 29·8%, exceeding the control rate in the same age group (16·9%) and the severe untreated prevalence in the working-age comparator (50–54 years: 26·6%) (Fig 2B; Table 1). Because severe untreated hypertension can reflect multiple points of failure (initiation, access, adherence, follow-up, or treatment intensification), we interpret this indicator as a public-health risk marker rather than a direct measure of clinician behaviour or treatment intensification.

### Geographic heterogeneity in age-related gaps

**Fig 3:**
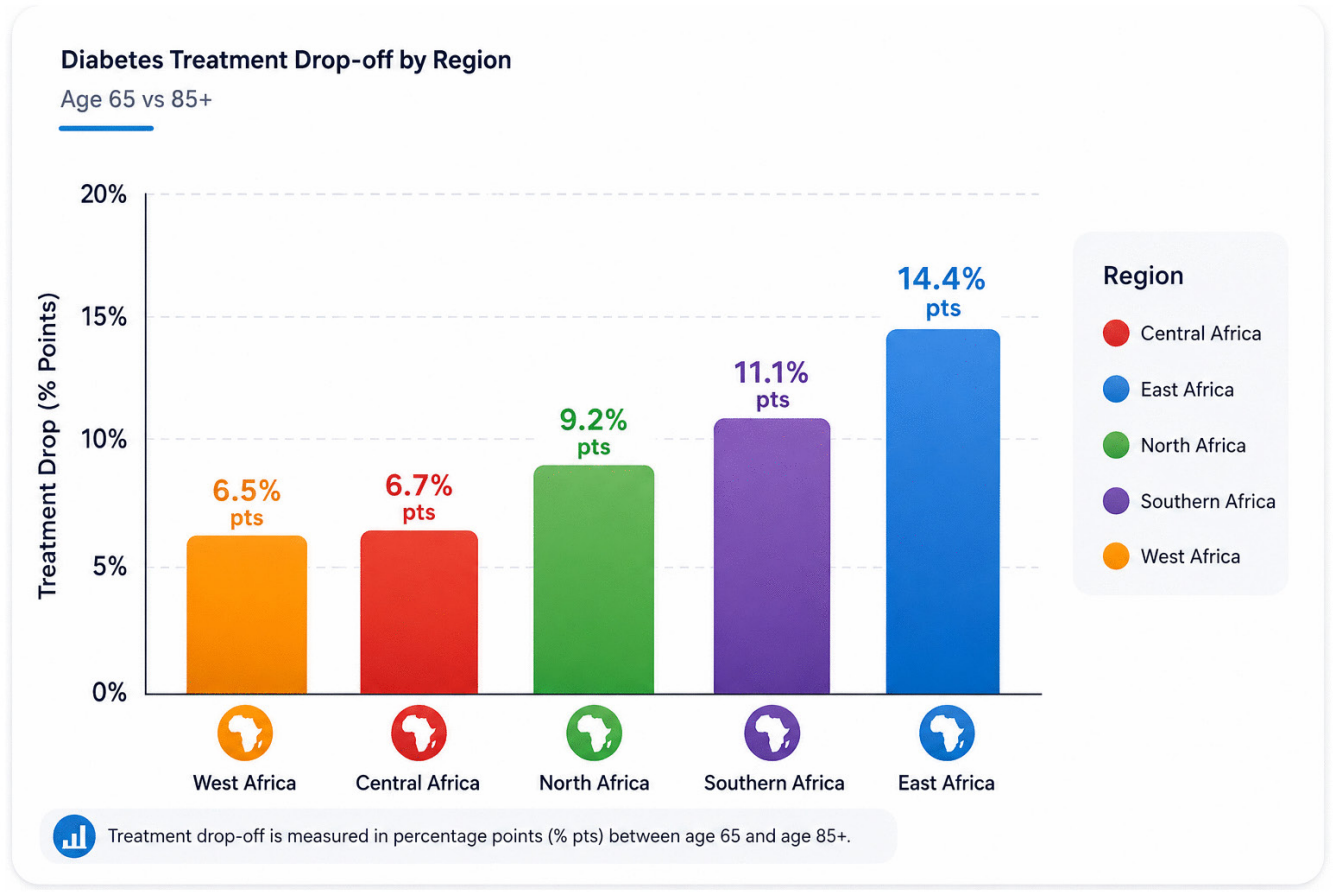
Regional differences in diabetes treatment coverage by age (2022). Posterior mean treatment coverage at 65–69 years versus ≥85 years, and the resulting age-related difference, by African Union region.

Age-related gaps were not geographically uniform. For diabetes, point-estimate differences in treatment coverage between ages 65–69 and ≥85 varied by subregion (Fig 3; Table 2). Interval-based aggregation indicated that the post-70 drop-off was most robust in East Africa, where the 95% uncertainty interval for the regional drop-off did not include zero (95% UI 6·6 to 19·1). In other regions, treatment coverage also tended to be lower at ≥85, but uncertainty intervals for the aggregated regional differences overlapped zero, consistent with wider uncertainty in the oldest age band and heterogeneous country patterns.

**Table 2:** Posterior mean treatment coverage at ages 65–69 and ≥85 years, and the age-related difference (65–69 minus ≥85), by African Union region.

African Union Region	Coverage (65–69)	Coverage (85+)	Drop-off Score	Interpretation
East Africa	37.3%	22.8%	14.4% pts	Largest age-related drop in coverage.
Southern Africa	30.9%	19.8%	11.1% pts	Large age-related drop in coverage.
North Africa	58.6%	49.4%	9.2% pts	Highest absolute coverage; age gradient persists.
Central Africa	24.7%	18.0%	6.7% pts	Low coverage at both ages; smaller drop reflects low baseline.
West Africa	26.2%	19.7%	6.5% pts	Low coverage at both ages; modest age-related drop.

**Fig 4:**
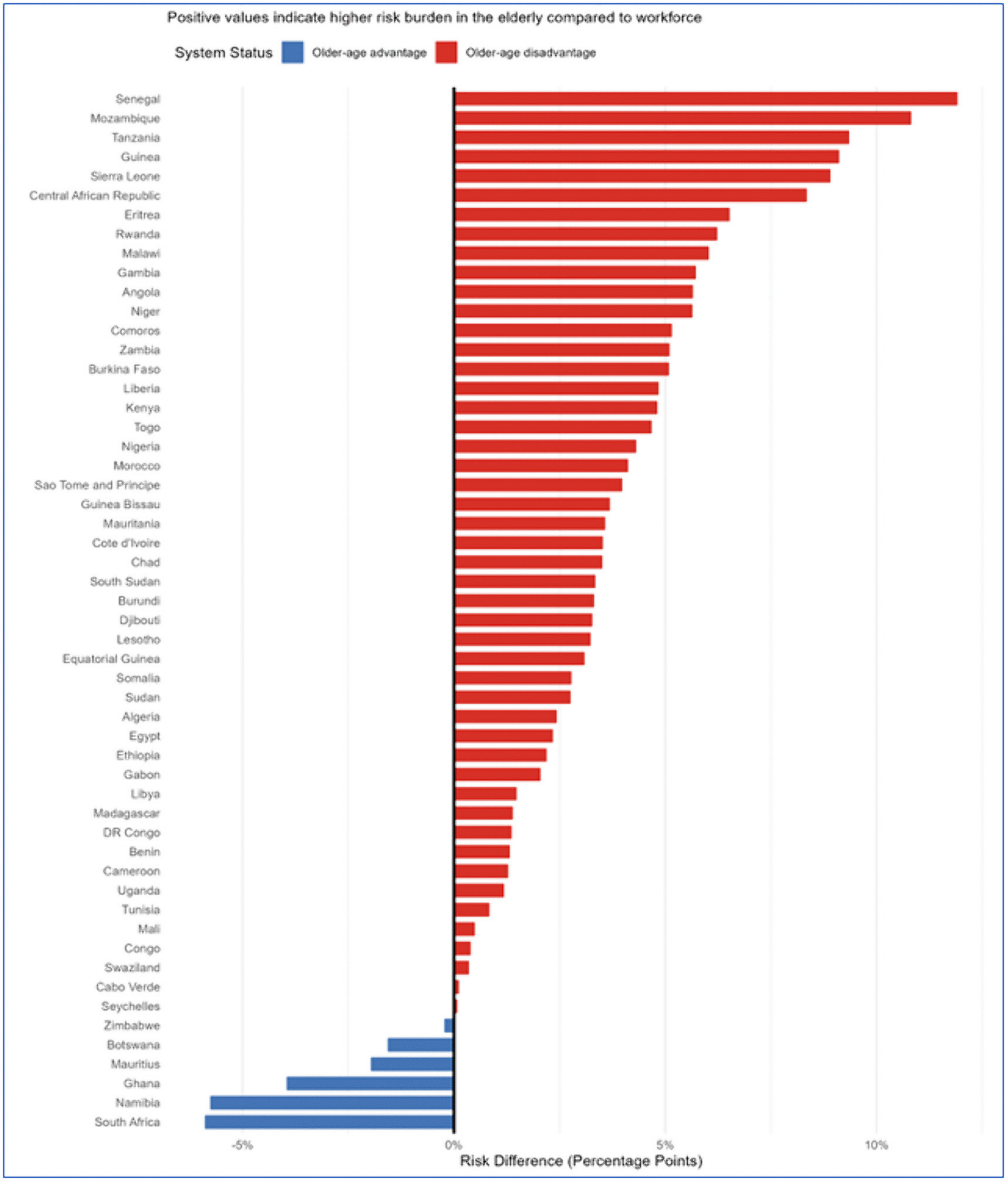
Disparity in untreated severe hypertension (2019): difference in the prevalence of untreated Stage 2 hypertension (BP > 160/100 mmHg) comparing older adults (75–79) with the workforce comparator (50–54) by country. Positive values indicate a higher, severe, untreated burden in older adults.

**Fig 5:**
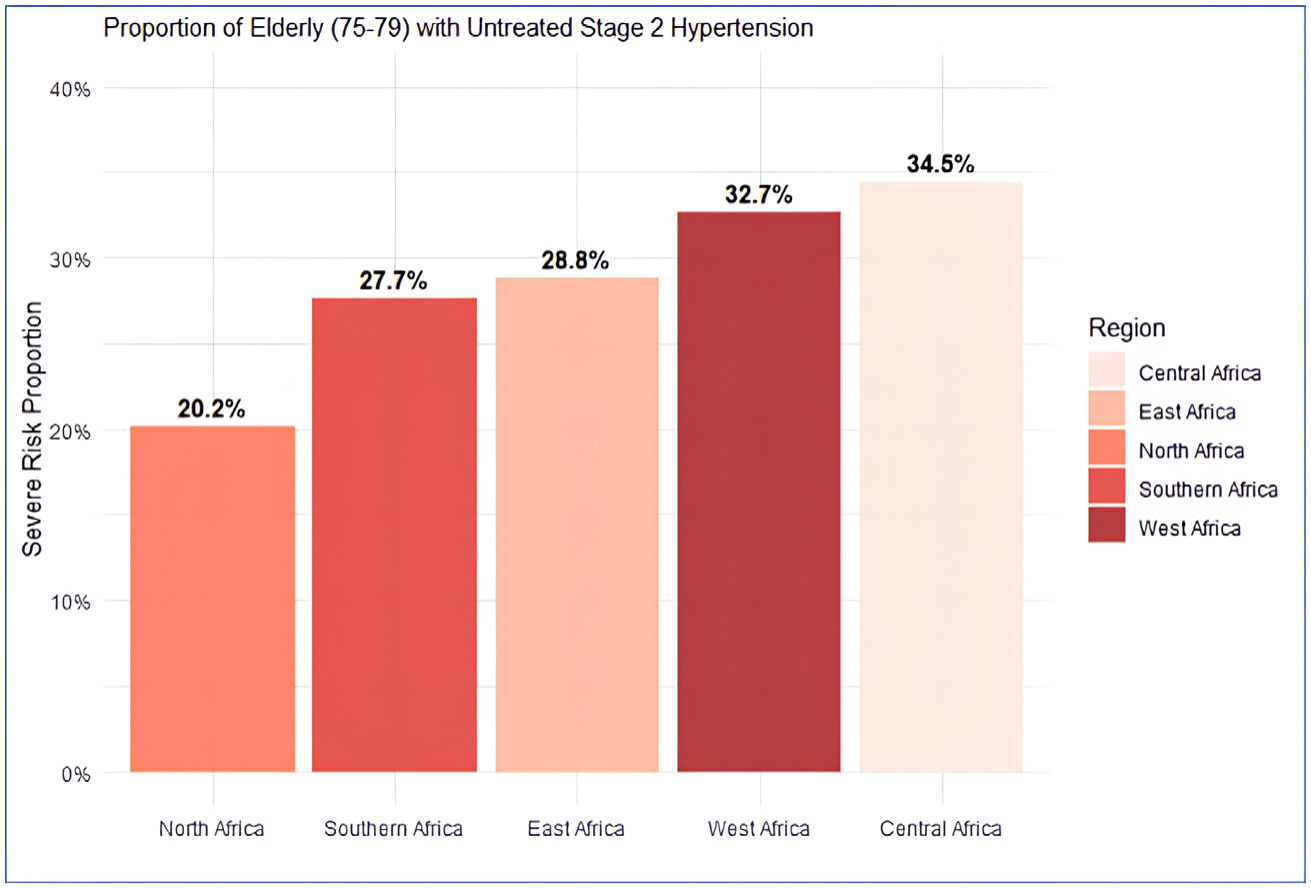
Severe untreated stage 2 hypertension among older adults (2019). Proportion of adults aged 75–79 years living with untreated stage 2 hypertension (BP > 160/100 mmHg) by African Union region.

For hypertension, most individual countries showed higher severe untreated stage 2 prevalence in older adults (75–79 years) than in the workforce comparator (50–54 years) (Fig 4). Regionally, severe untreated stage 2 prevalence among adults aged 75–79 years was highest in Central Africa (34·5%) and West Africa (32·7%) (Fig 5; Table 3).

**Table 3:** Proportion of the older adult population living with untreated severe hypertension (BP > 160/100 mmHg).

Region	Prevalence (Burden)	Untreated Stage 2 (Risk)	Status
Central Africa	66.7%	34.5%	Highest severe untreated stage 2 prevalence.
West Africa	62.4%	32.7%	High severe untreated stage 2 prevalence.
East Africa	60.3%	28.8%	Moderately high severe untreated stage 2 prevalence.
Southern Africa	67.2%	27.7%	Lower severe untreated stage 2 prevalence than Central/West.
North Africa	72.9%	20.2%	Lowest severe untreated stage 2 prevalence.

Tunisia was an outlier with comparatively small age differences in diabetes treatment coverage across older age bands (Fig 7C). This descriptive finding suggests that smaller age gradients are achievable within the region, but the underlying system features require further investigation.

**Fig 6:**
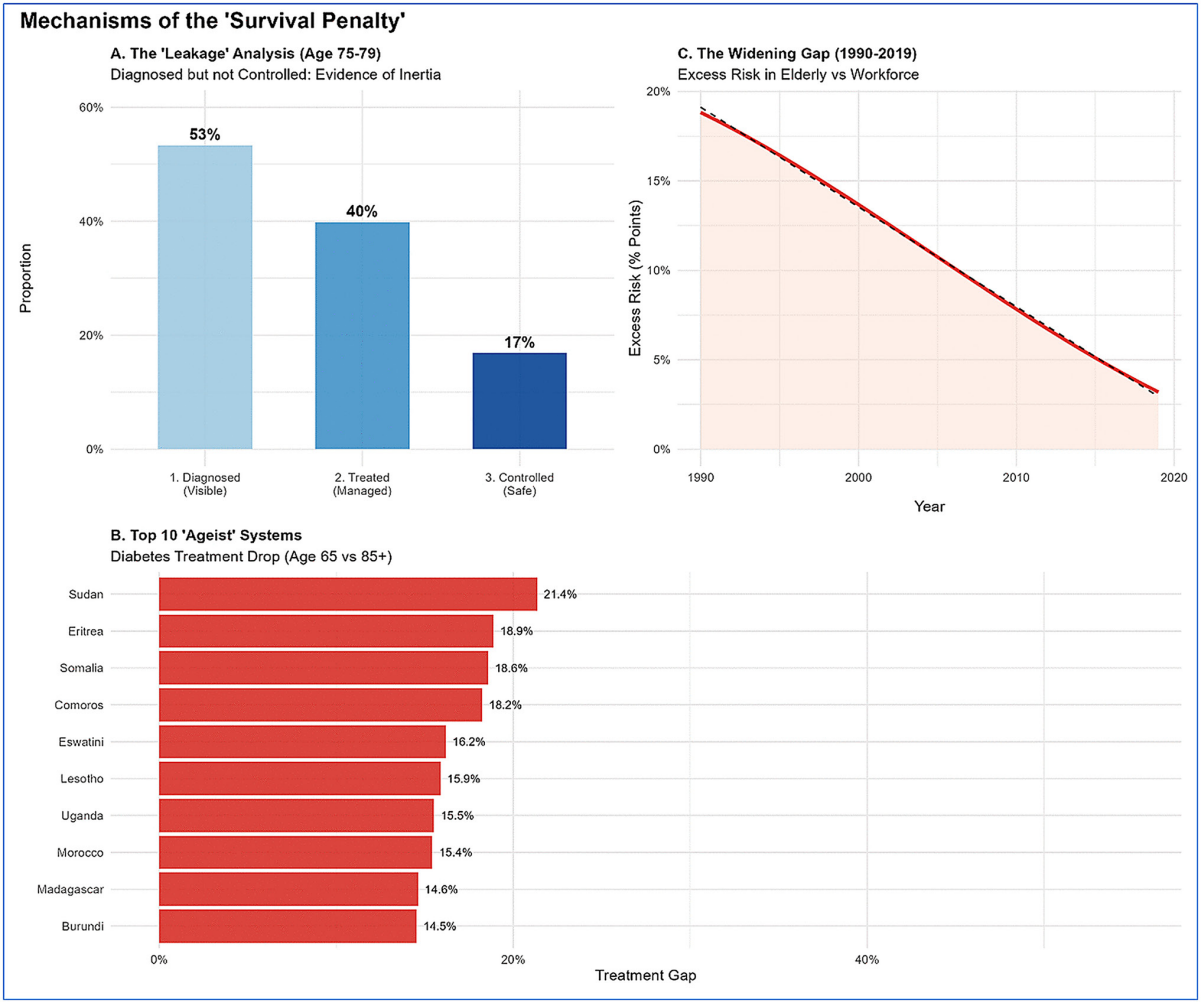
Cascade gaps and temporal dynamics. (A) Hypertension cascade indicators in older adults (75–79): proportions diagnosed, treated, and controlled. (B) Top 10 countries with the largest diabetes treatment coverage difference between 65–69 and ≥85. (C) Longitudinal trajectory (1990–2019) of the elderly–workforce difference in severe untreated stage 2 hypertension.

**Fig 7:**
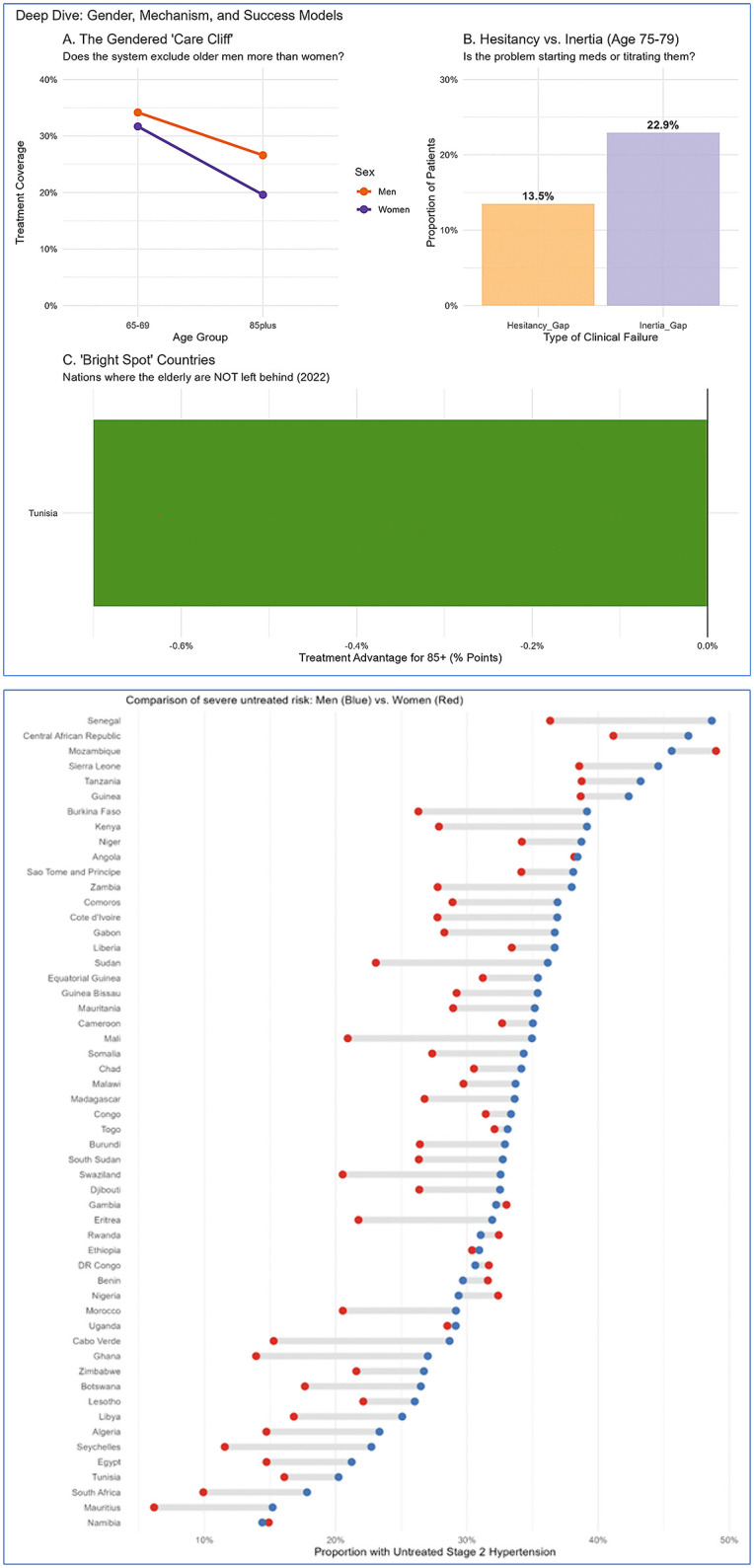
A–C Modifiers and potential success models. ***(A)*** *Sex-stratified diabetes treatment coverage at 65–69 vs ≥ 85.* ***(B)*** *Initiation versus downstream management gaps in older adults (75–79): diagnosed but untreated versus treated but uncontrolled, presented as descriptive components of cascade shortfalls.* ***(C)*** *Countries showing comparatively small age differences (or parity) for ≥85 in diabetes treatment coverage.* Sex difference in untreated Stage 2 hypertension (75–79): country-level comparison of severe untreated risk in men vs women.

### Mechanisms and temporal trends

Cascade patterns indicated that age-related differences were most pronounced downstream of diagnosis for hypertension: among older adults diagnosed with hypertension, a substantial proportion were treated but not controlled (Fig 6A). For diabetes, country-level point estimates showed marked variation in the 65–69 versus ≥85 treatment coverage difference (Fig 6B); however, country-level inference is limited by wide uncertainty in the ≥ 85 estimates. Longitudinally, the elderly–workforce difference in severe untreated stage 2 hypertension increased from 14.6 percentage points in 1990 to 21.8 in 2019 (Fig 6C).

### Modifiers

Gender-stratified analyses revealed heterogeneous patterns. Sex differences in the diabetes treatment coverage difference suggested that age gradients may not be uniform for men and women (Fig 7A), while country-level data showed substantial variation in severe untreated stage 2 hypertension by sex (Fig 7D). Analyses comparing initiation and downstream management gaps suggest that observed control differences at older ages are more consistent with downstream management shortfalls (treated but uncontrolled) than with low treatment initiation alone, while recognising that these components cannot be causally separated using aggregated data (Fig 7B).

## Discussion

Our ecological analysis of age-stratified, modelled estimates suggests that African health systems exhibit patterns consistent with a systematic “survival penalty” in the management of non-communicable diseases (NCDs), whereby the intensity of care appears to weaken with advancing age despite rising disease burden. Older adults carried a substantially higher prevalence of hypertension yet achieved no meaningful advantage in control, creating a diagnostic paradox in which increased detection failed to translate into protection from severe clinical risk. For diabetes, treatment coverage peaked in the “young-old” and declined sharply in the oldest-old age groups, revealing an age-related reduction in service intensity that is not aligned with expected clinical need. These patterns were not uniform but clustered geographically, with East and Southern Africa showing the greatest shortfalls in diabetes treatment while Central and West Africa carried the highest burden of severe untreated hypertension. Longitudinally, these age-related inequities appear to have widened since 1990, indicating that health system gains may have disproportionately benefited younger cohorts. These findings point to age-based inequities as a potentially structural feature of African NCD systems, rather than a transient anomaly.

The diagnosis–control gap observed for hypertension indicates that increased diagnostic contact at older ages does not necessarily translate into sustained control. Several explanations are plausible, including inconsistent medication availability, episodic follow-up, suboptimal titration, and adherence barriers [12,13]. In addition, treatment targets and therapeutic aggressiveness may differ for frail older adults because of polypharmacy, orthostatic hypotension, and competing risks [14–16]; therefore, low control should not be interpreted as inappropriate care in all cases. Nonetheless, persistent severe untreated hypertension suggests that avoidable risk remains substantial in many settings.

For diabetes, treatment coverage showed a marked post-70 decline when comparing ages 65–69 with ≥85. This pattern could reflect a combination of factors, including access and affordability barriers, differential clinical targets in very old age, and survivorship bias (healthy survivor effect), whereby older age groups represent a selected subset of individuals who have survived earlier mortality risks, as well as measurement limitations in the underlying surveys [17,18]. This may also contribute to the observed lower prevalence of diabetes in the ≥ 85 cohort. In the absence of explicit geriatric guidelines and robust trial evidence, declining diabetes treatment at advanced ages may also be consistent with clinical decision-making under uncertainty regarding benefit in very old patients, including potential therapeutic inertia [19,20]. The ecological design cannot distinguish among these explanations, but the magnitude and consistency of the decline across multiple settings warrants attention as a potential equity and service-delivery issue.

Severe untreated stage 2 hypertension among adults aged 75–79 years remains highly prevalent in Central and West Africa, reflecting gaps in treatment and follow-up. Given that severe hypertension is a modifiable risk factor for stroke and heart failure and that intensive control yields substantial risk reduction, this burden is particularly concerning [21–23]. Importantly, severe untreated burden is compatible with multiple cascade failures (non-initiation, interruption of therapy, inadequate intensification, or limited follow-up), emphasising that detection alone is an incomplete indicator of system performance [24].

The pronounced regional gradients argue against a purely biological explanation and instead point to health system factors as likely contributors to the observed differences, although causal attribution cannot be established. North Africa, with higher treatment coverage and lower severe untreated burden, resembles more developed chronic care models, whereas other regions show patterns consistent with gaps in access and continuity of care, particularly at older ages [25]. Tunisia’s comparatively small age differences in diabetes treatment coverage highlight that smaller age gradients are achievable within Africa, although further comparative work is needed to identify the specific policies or delivery features associated with this pattern [26].

The widening gap in untreated severe hypertension between older adults and the working-age population suggests that age-related inequities have not narrowed over time and may be increasing. This pattern is consistent with health system improvements disproportionately benefiting younger cohorts, leaving the oldest-old relatively underserved, a dynamic aligned with the concept of stratified universalism [27]. However, these trends should be interpreted alongside potential influences of data availability, model performance, and survivorship effects, and warrant confirmation using complementary data sources.

A major strength of this study is the use of the largest harmonised dataset of age-stratified NCD care cascades available for Africa, covering all 54 African Union member states over three decades. The use of nationally representative, model-based estimates allows for direct comparison across countries and time. Nonetheless, certain limitations should be taken into account. Our analysis relies on aggregated modelled data rather than individual-level clinical records, which precludes adjustment for comorbidities, frailty, or treatment adherence and service availability. Additionally, the absence of an explicit 80 + hypertension stratum required the use of the 75–79 group as a proxy, which may underestimate age-related differences in hypertension indicators. Differences in upper age strata across conditions may also limit direct comparability of estimates. Finally, diagnostic and treatment definitions reflect available survey data rather than guideline-concordant clinical care, and uncertainty for derived estimates was propagated from published 95% intervals under assumptions of independence, such that correlations within the underlying models may influence the precision of reported differences.

For practice, these findings highlight the need to manage older adults with NCDs as a high-risk group requiring proactive titration and continuity rather than passive maintenance. Clinical guidelines should explicitly include age-specific escalation protocols to counter therapeutic inertia. For policy, UHC frameworks must move beyond simple enrolment metrics toward equity-sensitive performance measures that track severe untreated risk in older adults. Financing packages should explicitly protect the oldest-old from potential age-related disparities in access to care. Future research should integrate longitudinal cohort data to identify the specific mechanisms underlying observed age-related differences and evaluate positive-deviant systems, such as Tunisia, to inform the design of age-equitable chronic care models in resource-constrained settings while triangulating findings with complementary data sources where possible.

## Conclusion

In conclusion, current African health systems appear to exhibit patterns consistent with a structural “survival penalty,” where the dividends of increased longevity are not consistently matched by sustained access to effective chronic care for the oldest-old. The divergence between high diagnostic visibility and low levels of disease control in hypertension, coupled with lower treatment coverage among older adults in diabetes care, indicates that Universal Health Coverage frameworks may be associated with age-related differences in care delivery across the life course. These findings challenge the assumption that expanding health access for the general population will automatically benefit geriatric cohorts; instead, they suggest a misalignment in which care intensity does not consistently correlate with clinical need across the life course. Without deliberate policy intervention to integrate geriatric-competent care into primary health platforms, the rising tide of NCDs risks disproportionately affecting the continent’s most vulnerable elders. Achieving true equity requires a more explicit and intentional redesign of service delivery, to ensure that the right to health does not diminish with advancing age.
