# Supplementary material for: The survival penalty: Age-based inequities in hypertension and diabetes management in African health systems (1990–2022) – a secondary quantitative analysis
Source: PLOS Glob Public Health. 2026 Jul 23;6(7):e0006306. doi: 10.1371/journal.pgph.0006306 (PMC13395376; doi:10.1371/journal.pgph.0006306)
Supplement: Attachment — Submitted filename: Response to Reviewers PLOS - R1.docx [file pgph.0006306.s002.docx]

**Response to Reviewers**

**Manuscript: “The survival penalty: age-based inequities in hypertension and diabetes management in African health systems (1990–2022)”**

**Journal: PLOS (Revised submission)**

**Overview of major revisions**

We thank the Academic Editor and all reviewers for their careful, constructive feedback. We have revised the manuscript substantially to align interpretation with the ecological, modelled nature of the NCD-RisC estimates, tighten operational definitions, improve transparency, and strengthen the reporting of uncertainty for derived metrics.

- Reframed the manuscript to report descriptive age-related gradients (rather than intent- or mechanism-implying language).
- Removed inferential p-value framing based on posterior means; emphasized effect sizes and uncertainty intervals where available.
- Added uncertainty propagation for the diabetes age-gap metric (“treatment coverage at 65–69 minus ≥85 in 2022”) using published 95% uncertainty intervals (UIs) and delta-method SE reconstruction; prioritised regional/continental pooled estimates where uncertainty is smaller.
- Clarified age-band definitions and comparators, explicitly tying them to available NCD-RisC age strata and noting comparability limits.
- Separated and clarified outcome constructs (treatment coverage gap vs hypertension cascade indicators vs severe untreated stage 2 burden).
- Updated data/code sharing statement; analysis code and derived-metric definitions are now available in Zenodo (DOI: 10.5281/zenodo.19337808).

Below, we provide a point-by-point response to editor’s and each reviewer’s comment, indicating where changes were made in the revised manuscript (by section). Within the manuscript, changes made are highlighted in red.

**Editor:**

| **Editor’s Requirement** | **Author’s Response** | **Location in Revised Manuscript** |
| --- | --- | --- |
| 1. Ethics Statement Placement & Content: Ensure the statement is at the beginning of the Methods section under a specific subheading and includes IRB details and consent status. | We have relocated the Ethics Statement to the very beginning of the Methods section. The statement now explicitly identifies the study as a secondary analysis of anonymized data and explains that formal consent was not obtained due to the nature of the aggregated data. | Methods (Ethics Statement) |
| 2. Figure File Formats: Provide separate figure files in .tif or .eps format. | All figures have been converted to .tif format and processed through the PLOS NAAS tool to ensure compliance with resolution and print-size requirements. | Submitted as separate files (Fig 1.tif through Fig 7.tif) |
| 3. Data Availability Policy: Ensure all data underlying findings are available in a public repository. | We have updated the Data Sharing statement to reflect that the analysis code and derived-metric definitions (e.g., Geriatric Drop-off Score) are now available in a permanent public repository via Zenodo (DOI: 10.5281/zenodo.19337808). | Declarations (Data sharing) |
| 4. Reporting Standards: Ensure Methods include clear mapping to STROBE/GATHER items. | We have explicitly cited adherence to GATHER and STROBE guidelines in the study design section. | Methods (Study Design) |
| 5. Reference Formatting: Correct formatting and numbering errors in the bibliography. | The reference list has been audited for Vancouver-style consistency, including the removal of erroneous spacing in journal abbreviations and the addition of mandatory [Accessed] dates for web-based sources. | References |

**Reviewer 1:**

We address each major issue raised as follows:

| **Reviewer comment** | **Response** | **Location in revised manuscript** |
| --- | --- | --- |
| Claims exceed what the data/design can support (mechanism/intent language). | We removed language implying intent or discrimination (e.g., “active exclusion,” “withdrawal,” “implicit rationing,” “health system ageism”) and reframed the main findings as descriptive age-related gradients in cascade indicators. Where interpretive hypotheses are discussed, we now explicitly label them as hypothesis-generating and acknowledge alternative explanations (clinical targets in frailty, adherence, selective survival, measurement limits). | Abstract; Research-in-context; Introduction; Results; Discussion; Conclusion |
| Uncertainty is not propagated; inferential testing is not well justified. | We removed p-value–based inference from posterior means and incorporated uncertainty propagation for derived differences using published 95% uncertainty intervals (UIs) from NCD-RisC. Specifically, for the diabetes age-gap metric (treatment coverage 65–69 minus ≥85 in 2022), we reconstructed SEs from UIs and propagated uncertainty into the gap estimate. Because country-level uncertainty is widest at ≥85, we report interval-based results primarily at regional and continental levels. Continental drop-off: 9.8 percentage points (95% UI 5.9 to 13.7). East Africa regional drop-off: 12.9 (95% UI 6.6 to 19.1). We also added a transparent description of the independence assumption (conservative) and its implications. | Methods (Statistical analysis—Uncertainty propagation); Results (Diabetes age gradients; Geographic heterogeneity); Limitations |
| Age-band definitions and comparators are inconsistent and appear ad hoc. | We clarified that age strata differ because the hypertension cascade and diabetes treatment datasets provide different upper age groupings. We now define the ‘oldest available’ strata as 75–79 for hypertension and ≥85 for diabetes and explicitly state the resulting limitation for cross-disease comparability. We also clarified the rationale for selecting 50–54 as a stable late-career working-age reference group for cross-country comparison. To reduce reliance on a single cut-point, we also present treatment coverage across all available age bands (Figure 2A), showing that the decline after age 70 is visible across the ordered age series and is not an artefact of the 65–69 vs ≥85 comparison alone. | Methods (Procedures and definitions); Strengths & Limitations |
| Operational definitions need tightening (inertia vs untreated stage 2). | We agree and revised terminology so that “severe untreated stage 2 hypertension” is treated as a public-health risk indicator reflecting multiple failure points (access, initiation, adherence, follow-up), rather than a direct measure of clinician therapeutic inertia. We also separated constructs in the Results: (i) diabetes treatment-coverage drop-off (65–69 vs ≥85), (ii) hypertension cascade indicators (diagnosis/treatment/control), and (iii) severe untreated stage 2 burden. | Methods (Outcomes); Results (Hypertension cascade; Severe untreated stage 2); Discussion |
| Internal consistency of numbers/tables must be audited. | We conducted an internal audit of all reported values across Abstract/Results/Tables/Figures and harmonised the directionality of comparisons (e.g., ‘50–54 vs 75–79’ and ‘65–69 vs ≥85’). Where values appeared identical across indicators (e.g., workforce prevalence and diagnosis), we verified them against the extracted NCD‑RisC estimates and clarified the indicator labels/definitions to avoid misinterpretation. We also ensured all tables explicitly label the comparison direction (‘Workforce: x% vs Older age group: y%’). | Abstract; Results; Tables 1–3; Figure captions |
| Data/code availability does not meet typical PLOS expectations. | We updated the Data sharing statement to indicate that the analysis code and derived-metric definitions are available in a permanent public repository (Zenodo; DOI: 10.5281/zenodo.19337808). The public repository is available on Zenodo (DOI: 10.5281/zenodo.19337808). | Declarations—Data sharing |

**Reviewer 2:**

| **Reviewer comment** | **Response** | **Location in revised manuscript** |
| --- | --- | --- |
| Please discuss policy learning from Tunisia as a positive outlier and what policymakers should do to enable UHC to address age-related inequities. | We added a more cautious ‘positive deviance’ paragraph noting Tunisia’s comparatively small age differences in diabetes treatment coverage as a descriptive outlier, and framed this as hypothesis-generating. We also expanded policy implications to emphasise age-specific cascade monitoring and continuity-of-care features (medication availability, follow-up systems, risk-based management) and suggested comparative learning from countries with smaller age gradients. | Results (Positive deviance); Discussion (Policy implications) |

**Reviewer 3:**

| **Reviewer comment** | **Response** | **Location in revised manuscript** |
| --- | --- | --- |
| Adopt a more cautious observational tone given ecological, modelled data; clarify interpretative boundaries. | We revised the framing across sections to maintain an observational/descriptive tone, added explicit interpretive boundary statements, and reserved interpretation primarily for the Discussion with alternative explanations highlighted. | Abstract; Introduction; Results; Discussion; Limitations |
| Clarify newly introduced indicators and handling of modelled uncertainty; include robustness/sensitivity considerations if feasible. | We clarified the construction and interpretation of derived differences, and added an uncertainty-propagation subsection describing how 95% UIs were propagated into the diabetes age-gap metric and pooled at regional/continental levels to increase stability. We also acknowledge residual dependence between age-specific posteriors as a conservative limitation. | Methods (Statistical analysis—Uncertainty propagation); Results (Diabetes); Limitations |
| Minor stylistic adjustments and consistent formatting for figures/tables; strengthen references. | We made figure and table titles more descriptive and neutral, ensured in-text figure callouts and captions are self-contained, and adjusted terminology density to reduce rhetorical constructs. References were reviewed for consistency. | Results (Figure callouts/captions; Tables); References |

**Reviewer 4:**

| **Reviewer comment** | **Response** | **Location in revised manuscript** |
| --- | --- | --- |
| Replace causal/mechanistic concepts (e.g., ‘survival penalty,’ ‘diagnostic paradox,’ ‘care cliff’) with descriptive or hypothetical interpretations. | We retained the title term as a descriptive label but removed mechanistic claims and reframed findings as descriptive age gradients. We avoided causal interpretations and explicitly stated that mechanisms cannot be inferred from aggregated modelled data. | Abstract; Introduction; Results; Discussion; Conclusion |
| Justify differing ‘oldest-old’ definitions (hypertension 75–79 vs diabetes ≥85). | We clarified that these are the oldest available strata in each dataset, added a rationale tied to NCD-RisC reporting, and noted that this limits direct comparability between conditions. | Methods (Definitions); Limitations |
| Paired t-tests, normality assumptions, multiple comparisons; consolidate repeated sex stratification. | We removed p-value–centred inference based on paired t-tests from posterior means, emphasised effect sizes and uncertainty intervals where available, and streamlined the sex-stratified reporting for clarity. | Methods (Statistical analysis); Results (Modifiers) |
| Acknowledge clinical appropriateness in very old age (frailty/targets). | We added nuance in the Discussion and Limitations acknowledging that treatment targets and prescribing decisions may differ in frail older adults, while noting that severe untreated stage 2 burden remains clinically important. | Discussion; Limitations |

**Reviewer 5:**

| **Reviewer comment** | **Response** | **Location in revised manuscript** |
| --- | --- | --- |
| Clarify recruitment / inclusion / exclusion and missing data handling; strengthen transparency and reproducibility. | We clarified that this is a secondary analysis of publicly available, aggregated NCD-RisC estimates and that participant recruitment and consent were conducted within the underlying population-based surveys synthesised by NCD-RisC (not by our team). We also clarified that we used NCD-RisC country-year-age estimates without additional imputation, and expanded the data/code availability statement to meet PLOS expectations. | Methods (Study design; Data sources/handling); Declarations—Data sharing |
| Avoid causal claims in Results/Conclusions and support statements with literature. | We revised Results and Conclusion language to remain descriptive and added literature acknowledging alternative explanations and geriatric target-setting considerations. | Results; Discussion; Conclusion; References |

**Reviewer 6:**

| **Reviewer comment** | **Response** | **Location in revised manuscript** |
| --- | --- | --- |
| Provide a clear rationale for choosing 50–54 as reference; clarify differing ‘oldest-old’ thresholds. | We added an explicit rationale for the 50–54 reference group as a stable late-career working-age comparator and clarified that upper age strata differ by dataset availability (hypertension 75–79; diabetes ≥85), noting comparability limits. | Methods (Definitions); Limitations |
| Consider more robust inference (e.g., multilevel regression) and ensure uncertainty is reflected in comparisons. | Given the ecological design and modelled inputs, we prioritised descriptive estimates and uncertainty intervals for derived differences where available. We added interval-based (UI-propagated) results for the diabetes age-gap metric at regional/continental levels. We note that extensions using hierarchical regression frameworks could be explored in future work, but would require careful modelling of dependence and uncertainty beyond the scope of this descriptive paper. | Methods (Statistical analysis—Uncertainty propagation); Discussion (Limitations/Future research) |
| Clarify whether data were weighted; link indicators to systems responsiveness; avoid direct evidence claims of ‘ageism.’ | We clarified that we computed unweighted means across countries to provide equal country contribution in continental/regional summaries and avoided interpreting gradients as evidence of intent or discrimination. We strengthened the conceptual framing around system responsiveness by linking findings to age-specific cascade monitoring and continuity-of-care features. | Methods (Statistical analysis); Discussion (Policy implications) |
