# Supplementary material for: The survival penalty: Age-based inequities in hypertension and diabetes management in African health systems (1990–2022) – a secondary quantitative analysis
Source: PLOS Glob Public Health. 2026 Jul 23;6(7):e0006306. doi: 10.1371/journal.pgph.0006306 (PMC13395376; doi:10.1371/journal.pgph.0006306)
Supplement: Attachment — Submitted filename: Response to Reviewers PLOS - R2.docx [file pgph.0006306.s003.docx]

**RESPONSE TO REVIEWERS**

We thank the reviewer for their thoughtful and constructive comments. We have carefully revised the manuscript to ensure strict alignment between the data, study design, and interpretation. All changes have been incorporated in the revised manuscript and highlighted in red.

**Comment 1:**

The formulation “which likely leads to an underestimation of the true survival penalty” implies a mechanism not demonstrated by the study.

**Response:**

We agree and have revised this statement to a more neutral and descriptive formulation. The text now reads: “may underestimate age-related differences in hypertension indicators.” We have also reviewed the manuscript to ensure that similar causal phrasing has been replaced with language consistent with an ecological study design.

**Comment 2:**

The manuscript does not explicitly discuss survivorship bias.

**Response:**

We appreciate this important point. We have now added an explicit limitation in the Discussion section noting that: “observed age gradients may partly reflect survivorship bias (healthy survivor effect), as older age groups represent a selected subset of healthier survivors.”
We also clarify how this may influence observed patterns in disease prevalence and treatment coverage at advanced ages.

**Comment 3:**

Several statements remain overly interpretative (e.g., “implicit rationing”, “age-stratified systems”).

**Response:**

We agree and have revised these statements throughout the manuscript to avoid implying unmeasured mechanisms. Interpretations have been reframed in descriptive terms, for example:
“observed age-related differences in care cascade indicators suggest heterogeneity across age groups, without allowing inference on underlying mechanisms or appropriateness of care intensity.” We have systematically reviewed the Discussion and Conclusion sections to ensure consistency with this approach.

We thank the reviewer again for these insightful suggestions, which have strengthened the clarity and methodological rigor of the manuscript.

**Dr. Bruno Basil**

Corresponding Author
