# Supplementary material for: The survival penalty: Age-based inequities in hypertension and diabetes management in African health systems (1990–2022) – a secondary quantitative analysis
Source: PLOS Glob Public Health. 2026 Jul 23;6(7):e0006306. doi: 10.1371/journal.pgph.0006306 (PMC13395376; doi:10.1371/journal.pgph.0006306)
Supplement: Peer review history

# Peer review: Decision Letter 0

**DOI:** 10.1371/journal.pgph.0006306.r001

**Authors:** Emmanuel Olamijuwon

15 Mar 2026

PGPH-D-26-00316

The survival penalty: age-based inequities in hypertension and diabetes management in African health systems (1990–2022) – a secondary quantitative analysis.

PLOS Global Public Health

Dear Dr. Basil,

Thank you for submitting your manuscript to PLOS Global Public Health. After careful consideration, we feel that it has merit but does not fully meet PLOS Global Public Health’s publication criteria as it currently stands. Therefore, we invite you to submit a revised version of the manuscript that addresses the points raised during the review process.

Please submit your revised manuscript by Apr 29 2026 11:59PM. If you will need more time than this to complete your revisions, please reply to this message or contact the journal office at globalpubhealth@plos.org. When you're ready to submit your revision, log on to https://www.editorialmanager.com/pgph/ and select the 'Submissions Needing Revision' folder to locate your manuscript file.

Please include the following items when submitting your revised manuscript:

- A letter that responds to each point raised by the editor and reviewer(s). You should upload this letter as a separate file labeled 'Response to Reviewers'.
- A marked-up copy of your manuscript that highlights changes made to the original version. You should upload this as a separate file labeled 'Revised Manuscript with Track Changes'.
- An unmarked version of your revised paper without tracked changes. You should upload this as a separate file labeled 'Manuscript'.

Guidelines for resubmitting your figure files are available below the reviewer comments at the end of this letter.

We look forward to receiving your revised manuscript.

Kind regards,

Emmanuel Olamijuwon, Ph.D

Academic Editor

PLOS Global Public Health

**Journal Requirements:**

1. Please ensure that your Ethics Statement is available in its entirety at the beginning of your Methods section, under a subheading 'Ethics Statement'. It must include:

i) The name(s) of the Institutional Review Board(s) or Ethics Committee(s)

ii) The approval number(s), or a statement that approval was granted by the named board(s)

iii) (for human participants or donors) - A statement that formal consent was obtained (must state whether verbal/written) OR the reason consent was not obtained (e.g., anonymity).

2. Please provide separate figure files in .tif or .eps format.

For more information about figure files please see our guidelines:

https://journals.plos.org/globalpublichealth/s/figures

https://journals.plos.org/globalpublichealth/s/figures#loc-file-requirements

3. In the online submission form, you indicated that “The data that support the findings of this study are available from the NCD Risk Factor Collaboration (NCD-RisC) at www.ncdrisc.org. The specific age-stratified aggregate estimates for hypertension and diabetes used in this analysis are publicly available for download. No individual participant data were used. The R code used for the calculation of the Geriatric Drop-off Score and the analysis of therapeutic inertia is available from the corresponding author upon reasonable request.”.

All PLOS journals now require all data underlying the findings described in their manuscript to be freely available to other researchers, either

1. In a public repository,

2. Within the manuscript itself, or

3. Uploaded as supplementary information.

This policy applies to all data except where public deposition would breach compliance with the protocol approved by your research ethics board. If your data cannot be made publicly available for ethical or legal reasons (e.g., public availability would compromise patient privacy), please explain your reasons by return email and your exemption request will be escalated to the editor for approval. Your exemption request will be handled independently and will not hold up the peer review process, but will need to be resolved should your manuscript be accepted for publication. One of the Editorial team will then be in touch if there are any issues.

If the reviewer comments include a recommendation to cite specific previously published works, please review and evaluate these publications to determine whether they are relevant and should be cited. There is no requirement to cite these works unless the editor has indicated otherwise.

**Additional Editor Comments (if provided):**

[Note: HTML markup is below. Please do not edit.]

Reviewers' comments:

Reviewer's Responses to Questions

**Comments to the Author**

1. Does this manuscript meet PLOS Global Public Health’s publication criteria? Is the manuscript technically sound, and do the data support the conclusions? The manuscript must describe methodologically and ethically rigorous research with conclusions that are appropriately drawn based on the data presented.

Reviewer #1: No

Reviewer #2: Yes

Reviewer #3: Yes

Reviewer #4: Partly

Reviewer #5: Yes

Reviewer #6: Partly

**********

2. Has the statistical analysis been performed appropriately and rigorously?

Reviewer #1: No

Reviewer #2: Yes

Reviewer #3: Yes

Reviewer #4: Yes

Reviewer #5: Yes

Reviewer #6: No

**********

3. Have the authors made all data underlying the findings in their manuscript fully available (please refer to the Data Availability Statement at the start of the manuscript PDF file)?

The PLOS Data policy requires authors to make all data underlying the findings described in their manuscript fully available without restriction, with rare exception. The data should be provided as part of the manuscript or its supporting information, or deposited to a public repository. For example, in addition to summary statistics, the data points behind means, medians and variance measures should be available. If there are restrictions on publicly sharing data—e.g. participant privacy or use of data from a third party—those must be specified.

Reviewer #1: No

Reviewer #2: Yes

Reviewer #3: Yes

Reviewer #4: Yes

Reviewer #5: Yes

Reviewer #6: Yes

**********

4. Is the manuscript presented in an intelligible fashion and written in standard English?

PLOS Global Public Health does not copyedit accepted manuscripts, so the language in submitted articles must be clear, correct, and unambiguous. Any typographical or grammatical errors should be corrected at revision, so please note any specific errors here.

Reviewer #1: Yes

Reviewer #2: Yes

Reviewer #3: Yes

Reviewer #4: Yes

Reviewer #5: Yes

Reviewer #6: Yes

**********

5. Review Comments to the Author

Please use the space provided to explain your answers to the questions above. You may also include additional comments for the author, including concerns about dual publication, research ethics, or publication ethics. (Please upload your review as an attachment if it exceeds 20,000 characters)

**Reviewer #1:**  General assessment

This manuscript addresses an important and understudied question: whether chronic disease management for hypertension and diabetes differs systematically by age across African health systems. The continental scope and use of age-stratified care cascade estimates are potentially valuable, and the descriptive signal reported (declining treatment coverage/control at older ages) is policy-relevant. However, in its current form, the manuscript overinterprets modeled aggregate estimates and employs causal/intentional language that is not supported by the design. Substantial revisions are required before the conclusions are justified.

Major issues (must address)

1. Claims exceed what the data/design can support (mechanism/intent language).

The manuscript repeatedly uses terms implying intent or discrimination (e.g., “active exclusion,” “withdrawal,” “implicit rationing,” “health system ageism,” “survival penalty”). With ecological, modeled, aggregate NCD-RisC estimates, the study can robustly describe age-associated gradients in cascade indicators, but it cannot attribute intent, decision-making, or mechanisms (e.g., “exclusion” vs “appropriate deprescribing” vs selective survival vs measurement limitations).

Required revision: Reframe primary findings as descriptive age gradients/inequities. If terms like “exclusion” or “ageism” are retained, they must be explicitly defined as descriptive constructs, and the limits of inference must be stated clearly in Methods/Limitations.

2. Uncertainty is not propagated; inferential testing is not well justified.

The analysis uses posterior mean estimates from NCD-RisC and applies paired t-tests and p-values as if they were directly observed measurements. This ignores estimation uncertainty (credible intervals/posterior distributions) and complicates the interpretation of p-values when using model-based inputs and accounting for cross-country dependence.

Required revision: Incorporate uncertainty-aware comparisons (e.g., using posterior intervals or draws where available), and report effect sizes with uncertainty rather than relying on p-values. If inferential statistics are retained, the manuscript must justify the inferential target (e.g., country-level comparative statistics) and clarify the assumptions.

3. Age-band definitions and comparators are inconsistent and appear ad hoc.

Hypertension “oldest-old” is 75–79, whereas diabetes “oldest-old” is ≥85; the “workforce” comparator is 50–54 for hypertension, but diabetes comparisons center on 65–69 vs ≥85. This will be read as selective framing unless justified by data availability and sensitivity analyses.

Required revision: Provide a principled rationale tied to available age strata, and add sensitivity analyses using alternative age cut points or using age as an ordered series to test the “care cliff” hypothesis (e.g., segmented trend after age 70).

4. Operational definitions need tightening (inertia vs untreated Stage 2).

The manuscript defines “therapeutic inertia” as a diagnosis–control gap, but then uses “untreated Stage 2 hypertension” as the main indicator. Severe untreated hypertension reflects multiple failure points (non-initiation, access barriers, adherence, follow-up gaps) and is not synonymous with inertia among treated patients.

Required revision: Separate cascade failure mechanisms explicitly:

• diagnosed but not treated (initiation gap),

• treated but uncontrolled (intensification/management gap),

• severe untreated hypertension burden (public health risk indicator).

Use terminology consistent with what is actually measured.

5. Internal consistency of numbers/tables must be audited.

Several values appear inconsistent across text and tables (e.g., control rates by age group; prevalence and diagnosis appearing identical in Table 1 for the workforce). Reviewers will flag these as credibility issues.

Required revision: Reconcile all reported values across Abstract/Results/Tables/Figures; explicitly label the direction of comparisons (“Workforce: x% vs Oldest-old: y%”).

6. Data/code availability does not meet typical PLOS expectations.

While the NCD-RisC estimates are public, the manuscript states that the analysis code is available “upon reasonable request.”

Required revision: Provide public access to the R code and derived metrics definitions (e.g., OSF/Zenodo/GitHub with a DOI if possible), sufficient for reproduction.

Minor issues/clarifications

• Guideline/target interpretation in very old age: The Discussion should explicitly acknowledge that treatment targets and therapeutic aggressiveness may differ in frail older adults and that some proportion of lower coverage could reflect clinical appropriateness; then justify why the observed magnitude/pattern still warrants concern, especially for severe untreated hypertension.

• Terminology density: The manuscript introduces multiple coined phrases (“care cliff,” “severity trap,” “male malus,” “female decline,” “diagnostic paradox”). Consider reducing or consolidating to improve clarity and reduce rhetorical tone.

• Reporting standards: If claiming STROBE/GATHER adherence, ensure the Methods include clear mapping to these items (variables, uncertainty, analytical choices).

• References formatting: The reference list shows numbering/formatting errors and should be corrected.

Summary recommendation

The study question is compelling and could make a useful descriptive contribution. However, the manuscript requires major revision to align claims with the limitations of modeled aggregate data, incorporate uncertainty appropriately, justify age-band choices, clarify outcome definitions, and address internal inconsistencies and issues with reproducibility.

**Reviewer #2:** Given this is the first continental analysis to quantify age78 based inequities in NCD management across all 54 African Union member states, the findings were informative to highlight UHC expansion has disproportionately benefited the working-age population, while the oldest-old face a widening risk gap in severe, untreated hypertension, particularly in Central and West Africa. The evidence collectively suggests that current Universal Health Coverage (UHC) frameworks in Africa are functionally age-stratified. The assumption that expanding general access will automatically protect vulnerable geriatric populations is flawed. Without specific safeguards, health systems appear to ration care implicitly by age. However, current Universal Health Coverage (UHC) frameworks often prioritize the economic core of the population, potentially creating a "Survival Penalty," a systemic withdrawal of care intensity as patients age beyond the workforce. Further the report on Tunisia as highlighted in the supplemental analyses as an outlier that maintains near-parity in diabetes treatment coverage across older age bands suggesting that system design and sustained primary care contact can mitigate age-related disparities.

What would be best is to point out how other countries can learn from Tunisia and what health policy makers have to do further to enable UHC in its true sense to address the age related inequities in health care access.

**Reviewer #3:** Thank you for the opportunity to review this manuscript. The study addresses a highly relevant and timely topic in global public health, examining age-related inequities in the management of hypertension and diabetes across African health systems. The continental scope, use of harmonised large-scale data, and clear policy relevance represent important strengths. Overall, the manuscript is well written, analytically sound, and conceptually innovative, and it has strong potential to contribute meaningfully to the literature.

The comments below are intended to support further refinement of the manuscript and to help ensure alignment between interpretation, methodology, and journal expectations.

Major comments

1. Framing and interpretation

Given the ecological nature of the analysis and reliance on aggregated modelled data, some sections of the manuscript would benefit from slightly more cautious wording. In a few instances, the language may be interpreted as implying causal mechanisms or intentional system-level decision-making. I encourage the authors to consider reframing such statements using a more observational tone, ensuring that interpretations remain closely aligned with the study design.

It would also be useful to include a brief statement acknowledging the ecological scope of the analysis and its interpretative boundaries.

2. Abstract and Results

The abstract is clear and informative. Minor adjustments emphasizing quantitative findings and reducing conceptual terminology may further enhance clarity. Similarly, in the Results section, maintaining a descriptive approach and reserving interpretation primarily for the Discussion would improve structural consistency.

3. Methods clarity

The methodological approach is appropriate and generally well described. However, the manuscript would benefit from additional clarification regarding:

• the development and interpretation of newly introduced indicators;

• statistical handling of modelled estimates and associated uncertainty;

• justification for statistical approaches applied to aggregated data.

If feasible, including a brief statement on robustness or sensitivity considerations would strengthen methodological transparency.

4. Discussion and Conclusion

The Discussion is thoughtful and well contextualized within the existing literature. Minor restructuring to clearly separate principal findings, comparison with prior work, and limitations may improve readability. In addition, slightly moderating causal or normative language would help maintain scientific neutrality.

The conclusion effectively summarizes the implications of the study; however, a more neutral closing tone focused on implications and future research directions may better align with journal style.

5. Presentation and references

Figures and tables are informative and support the narrative well. Some minor stylistic adjustments toward a more neutral journal presentation (e.g., descriptive titles and consistent formatting) could improve presentation. Additionally, increasing reliance on peer-reviewed references where possible may further strengthen the manuscript.

Overall recommendation

This is a strong and important manuscript addressing a relevant global health issue. The suggested revisions are primarily related to clarification, tone, and presentation rather than major analytical concerns. I believe the manuscript will be substantially strengthened once these refinements are addressed.

**Reviewer #4:**  Title : The survival penalty: age-based inequities in hypertension and diabetes managementin African health systems (1990–2022) – a secondary quantitative analysis

Version : 05 Mars 2026

Reviewer : Paulin Nkolamoyo Musungula

Reviewer's report : This manuscript addresses a highly relevant issue in global health: age-related inequalities in the management of hypertension and diabetes within African health systems over an extended period (1990–2022). The choice of a secondary quantitative analysis spanning more than three decades represents a significant strength, as it enables the examination of structural trends and situates the question of inequities within a robust temporal perspective. The study has substantial scientific and programmatic potential, particularly in informing health equity policies and guiding the adaptation of health systems to ongoing demographic and epidemiological transitions.

Overall, this is a timely and relevant study that could make an important contribution to the debate on intergenerational equity in health in Africa. However, several sections require clarification, and the manuscript could be improved as follows :

Abstract

The abstract is clear and relevant. However, terms such as “survival penalty,” “diagnostic paradox,” “care cliff,” “active exclusion,” and “therapeutic inertia” imply causal mechanisms. Given that the analysis is based on aggregated and modeled data, it would be preferable to present these concepts as descriptive or hypothetical interpretations, ensuring alignment between the conclusions and the nature of the data.

Introduction

1. Lines 103–105: You present the concept of a “Survival Penalty” as a hypothesis, which is entirely legitimate and conceptually interesting. However, the wording “a systemic withdrawal of care intensity as patients age beyond the workforce” is relatively assertive and may give the impression that a structural mechanism has already been established. I would suggest clarifying more explicitly the exploratory nature of this proposition and adopting slightly more conditional language (for example, referring to a “possible association between advancing age and a reduction in the intensity of care”). Such a reformulation would preserve the conceptual strength of your hypothesis while avoiding any impression of a premature conclusion.

Methods

1. Lines 141–145: You define the “Oldest-Old” group differently depending on the disease (75–79 years for hypertension and ≥85 years for diabetes). This complicates comparability between the analyses. It would be helpful to justify this difference or, if possible, harmonize the thresholds to strengthen consistency.

2. Lines 146–147: You use the term “Health System Ageism,” which implies systemic discrimination. Given that your data are aggregated and modeled, it would be more cautious to adopt more descriptive language, such as “age-related disparities in care,” to more accurately reflect what is actually measured.

3. Lines 148–157: You introduce the “Geriatric Drop-off Score” and the concept of “Therapeutic Inertia.” These indicators are interesting, but their limitations should be specified, including: the influence of selective mortality, disease prevalence, the limitations of modeled data, and the fact that therapeutic inertia traditionally implies clinician behavior, which is not directly measured here.

4. Lines 164–173: You use paired t-tests with N = 54 countries and a threshold of p < 0.05. It would be useful to clarify the assumptions of normality and independence and to discuss the issue of multiple comparisons. In addition, sex stratification is repeated in two places and could be consolidated for greater clarity.

5. Lines 161–162 and 169–170: You refer to the “Male Malus” and “Female Decline” hypotheses. These concepts are exploratory and somewhat normative. Their exploratory nature should be explicitly stated to avoid any causal interpretation.

Results

1. Lines 185–191: You describe the “diagnostic paradox” for hypertension. It should be recalled that this term describes an observed phenomenon and does not imply causality.

2. Lines 193–198: The “care cliff” for diabetes shows a decline in coverage after age 70. It would be useful to remind readers that these observations are based on aggregated and modeled data and therefore do not allow inference about intentional actions by health systems.

3. Lines 200–206: The analysis of “untreated Stage 2 hypertension” and the concept of a “severity trap” are relevant, but it should be clarified that therapeutic inertia is inferred from the data and does not directly measure clinical practice.

4. Lines 222–231: The regional differences (“Exclusion vs. Neglect”) are interesting but should include a nuance regarding the variability in data quality and health system capacity across regions.

5. Lines 251–253: You highlight Tunisia as an example of “positive deviance.” It would be helpful to clarify that this is a descriptive observation and that structural or policy causes cannot be directly inferred.

6. Lines 256–263: The analysis of temporal trends (risk gap) is relevant, but it should be recalled that these measures are based on aggregated and modeled data.

7. Lines 270–288: The sex-specific analyses and the distinction between “therapeutic inertia” and “clinical hesitancy” are useful, but it should be emphasized that these categories are indirectly estimated and do not directly measure individual clinical behavior.

Discussion

The discussion is generally strong and relevant. However, terms such as “survival penalty,” “diagnostic paradox,” and “care cliff” should be used in a descriptive and hypothetical manner in order to avoid any causal or intentional interpretation of health system behavior, given the aggregated and modeled nature of the data used.

Strengths and Limitations

Although this study uses the largest harmonized dataset on NCD care in Africa, several limitations should also be acknowledged. The data are aggregated and modeled, derived from heterogeneous sources of varying quality, without individual cohort follow-up, and may be affected by selective mortality. The absence of individual clinical data prevents adjustment for comorbidities, frailty, or treatment adherence, and the inference of therapeutic inertia relies on observed gaps rather than directly measured behaviors. The use of different age thresholds for hypertension and diabetes limits comparability, and socioeconomic factors are not controlled for. Finally, the observed phenomena such as the “care cliff” or the “diagnostic paradox” should remain descriptive and hypothetical rather than being interpreted causally.

Conclusion

Lines 372–382: The terms “structural survival penalty” and “withdrawal of effective care” suggest intention or causality that has not been demonstrated. It would be preferable to reformulate them in a more descriptive and hypothetical manner, for example by referring to a “possible reduction in the intensity of care among the oldest age groups.”

**Reviewer #5:**  Review Comments to the Authors

The manuscript presents an original study looking at age-based differences in non-communicable disease (NCD) care across Africa. This is an important topic, and the study provides useful information that could help guide policy and improve care for older adults. With chronic conditions like diabetes, hypertension, and heart disease becoming more common in aging populations, understanding where care gaps exist is very valuable.

Significance

The topic is timely and important. The findings help us understand where health systems may not be serving older adults well. This could support programs and policies aimed at making care more fair and accessible, especially in rural areas or underserved communities.

Literature Context

The authors cite important studies, but some points could be better supported by linking to more research. For example, when discussing how older adults may be excluded from care or guidelines, citing studies on geriatric care or health inequities would help. Comparing the results with data from other countries or regions could also make the findings easier to interpret.

Methods

The study design is generally appropriate, but more detail would make it clearer. For example, it would help to explain how participants were recruited, what the inclusion or exclusion criteria were, and if there were any changes to the original plan. Adding the full protocol or data collection tools as supplemental material would increase transparency. Also, it would be good to explain how countries with missing data were handled, even if the NCD-RisC imputation did this automatically.

Data Availability

Summary statistics are given, which is good, but the full or de-identified datasets should be shared according to PLOS rules. If there are reasons why the data can’t be shared, those should be clearly stated. Sharing the data makes it easier for other researchers to verify or build on the work.

Results and Conclusions

Most conclusions match the data. However, because this is an observational study, the authors should be careful about making causal claims. For example, instead of saying “older adults get worse care because of systemic neglect,” it would be better to say “older adults were less likely to receive recommended care, which may reflect systemic barriers.” This keeps the conclusions accurate without over-interpreting the findings.

Reproducibility

The methods are mostly clear, but adding more details about the tools used, how data collectors were trained, and how missing data were handled would help others repeat the study. This makes the research more reliable and useful for future studies.

Ethics and Compliance

Ethics approval and consent are mentioned, which is good and shows that the study follows standard research practices.

Writing and Organization

The manuscript is readable and well-organized. Some small improvements could make it even better, like simplifying long sentences, using consistent terms throughout, and adding more details in figure and table captions so the results are easy to understand.

**Reviewer #6:**  The topic is highly relevant for global health systems research, particularly given rapid demographic aging and increasing NCD burden in low- and middle-income countries. The use of large harmonized datasets covering multiple decades is a major strength. The paper is conceptually interesting and potentially impactful. However, this paper needs substantial methodological and conceptual refinements.

I would like to have a clear understanding of the rationale for choosing the 50–54 age group as a reference rather than the full adult population.

It would be helpful to have some clarity on the rationale for the difference between hypertensive and diabetic patients under the “Oldest-Old” category. The difference in age groups between HTN and DM reduces the comparability.

Was the data weighted before being used for analysis?

Providing a clear conceptual framework on how these indicators map onto health system behavior may be helpful for readers.

It could be helpful for the reader if some of the interesting concepts, such as "survival penalty," “active exclusion,” and “therapeutic inertia,” are sufficiently grounded in existing literature or in a validated matrix.

Given the structure of the data set (number of countries, years, age group, etc.), having a multilevel regression analysis would provide more robust inference.

I think the author needs to be more cautious in case of framing the age-related disparities as direct evidence of ‘health system ageism.’

Is data on health financing, service availability, comorbidities, etc., available in this dataset? A conclusion regarding therapeutic inertia may sound overstated without including those variables.

Providing the confidence intervals/statistical comparisons for comparisons would make it more robust.

The discussion section is quite compelling in interpreting the age-related disparities in NCD care. However, I think there is still scope for explicit articulation of the link between the findings of this manuscript and the broader concept of systems responsiveness.

There are lots of strengths of this study, like a large data set, a longitudinal perspective, and a focus on aging and NCDs. Clarifications on methodological aspects and conceptual refinement are necessary for the robustness of the study.

**********

6. PLOS authors have the option to publish the peer review history of their article (what does this mean?). If published, this will include your full peer review and any attached files.

**Do you want your identity to be public for this peer review?**  If you choose “no”, your identity will remain anonymous but your review may still be made public.

For information about this choice, including consent withdrawal, please see our Privacy Policy.

Reviewer #1: **Yes:** Abimbola Adegoke

Reviewer #2: **Yes:** Dr. Somasundari Gopalakrishnan

Reviewer #3: **Yes:** ALESSANDRA COELHO VIVEKANANDA MEIRELES

Reviewer #4: No

Reviewer #5: **Yes:** Neema W. Machumu

Reviewer #6: No

**********

[NOTE: If reviewer comments were submitted as an attachment file, they will be attached to this email and accessible via the submission site. Please log into your account, locate the manuscript record, and check for the action link "View Attachments". If this link does not appear, there are no attachment files.]

Figure Resubmissions:

While revising your submission, we strongly recommend that you use PLOS’s NAAS tool (https://ngplosjournals.pagemajik.ai/artanalysis) to test your figure files. NAAS can convert your figure files to the TIFF file type and meet basic requirements (such as print size, resolution), or provide you with a report on issues that do not meet our requirements and that NAAS cannot fix.

After uploading your figures to PLOS’s NAAS tool - https://ngplosjournals.pagemajik.ai/artanalysis, NAAS will process the files provided and display the results in the "Uploaded Files" section of the page as the processing is complete. If the uploaded figures meet our requirements (or NAAS is able to fix the files to meet our requirements), the figure will be marked as "fixed" above. If NAAS is unable to fix the files, a red "failed" label will appear above. When NAAS has confirmed that the figure files meet our requirements, please download the file via the download option, and include these NAAS processed figure files when submitting your revised manuscript.

# Author comment: Author response to Decision Letter 1

**DOI:** 10.1371/journal.pgph.0006306.r002

31 Mar 2026

Attachment Submitted filename: Response to Reviewers PLOS - R1.docx

# Peer review: Decision Letter 1

**DOI:** 10.1371/journal.pgph.0006306.r003

**Authors:** Priyanka Roy

16 Jun 2026

PGPH-D-26-00316R1

The survival penalty: age-based inequities in hypertension and diabetes management in African health systems (1990–2022) – a secondary quantitative analysis.

PLOS Global Public Health

Dear Dr. Basil,

Thank you for submitting your manuscript to PLOS Global Public Health. After careful consideration, we feel that it has merit but does not fully meet PLOS Global Public Health’s publication criteria as it currently stands. Therefore, we invite you to submit a revised version of the manuscript that addresses the points raised during the review process.

Please submit your revised manuscript by Jul 16 2026 11:59PM. If you will need more time than this to complete your revisions, please reply to this message or contact the journal office at globalpubhealth@plos.org. When you're ready to submit your revision, log on to https://www.editorialmanager.com/pgph/ and select the 'Submissions Needing Revision' folder to locate your manuscript file.

Please include the following items when submitting your revised manuscript:

- A letter that responds to each point raised by the editor and reviewer(s). You should upload this letter as a separate file labeled 'Response to Reviewers'.
- A marked-up copy of your manuscript that highlights changes made to the original version. You should upload this as a separate file labeled 'Revised Manuscript with Track Changes'.
- An unmarked version of your revised paper without tracked changes. You should upload this as a separate file labeled 'Manuscript'.

Guidelines for resubmitting your figure files are available below the reviewer comments at the end of this letter.

As the corresponding author, your ORCID iD is verified in the submission system and will appear in the published article. PLOS supports the use of ORCID, and we encourage all coauthors to register for an ORCID iD and use it as well. Please encourage your coauthors to verify their ORCID iD within the submission system before final acceptance, as unverified ORCID iDs will not appear in the published article. *Only*  the individual author can complete the verification step; PLOS staff *cannot* verify ORCID iDs on behalf of authors.

We look forward to receiving your revised manuscript.

Kind regards,

Priyanka Roy, MD, MPH, PhD

Academic Editor

PLOS Global Public Health

Journal Requirements:

If the reviewer comments include a recommendation to cite specific previously published works, please review and evaluate these publications to determine whether they are relevant and should be cited. There is no requirement to cite these works unless the editor has indicated otherwise.

Please review your reference list to ensure that it is complete and correct. If you have cited papers that have been retracted, please include the rationale for doing so in the manuscript text, or remove these references and replace them with relevant current references. Any changes to the reference list should be mentioned in the rebuttal letter that accompanies your revised manuscript. If you need to cite a retracted article, indicate the article’s retracted status in the References list and also include a citation and full reference for the retraction notice.

Additional Editor Comments (if provided):

Minor changes

Reviewer's report: The authors have overall responded in a thorough and satisfactory manner to all methodological comments. Without questioning the overall quality of the work, I remain nevertheless concerned about several points related to interpretation and wording, which would benefit from adjustment in order to ensure strict alignment between the data and the conclusions.

1. The following formulation remains problematic: “which likely leads to an underestimation of the true survival penalty (Line 370)”. It implicitly assumes the existence of a mechanism (“survival penalty”) that is not demonstrated by the study. A more neutral formulation would be preferable, for example: “may underestimate age-related differences in hypertension indicators”, in order to remain strictly descriptive and consistent with an ecological study design.

2. The manuscript does not explicitly discuss survivorship bias (healthy survivor effect), which is nevertheless essential for the interpretation of older age groups (75–79 years and ≥85 years). An appropriate correction would be to add an explicit limitation such as: “observed age gradients may partly reflect survivorship bias, as older age groups represent a selected subset of healthier survivors”.

3. Several statements remain overly interpretative, in particular: “implicit rationing (Line 381)”, “systems functioning in ways that are implicitly age-stratified (Line 392)”, and “care intensity does not consistently correlate with clinical need (Line 394)”. These formulations imply unmeasured mechanisms. A more rigorous version would be to rephrase in terms of descriptive associations, for example: “observed age-related differences in care cascade indicators suggest heterogeneity across age groups, without allowing inference on underlying mechanisms or on the appropriateness of care intensity”.

[Note: HTML markup is below. Please do not edit.]

Reviewers' comments:

Reviewer's Responses to Questions

**Comments to the Author**

1. If the authors have adequately addressed your comments raised in a previous round of review and you feel that this manuscript is now acceptable for publication, you may indicate that here to bypass the “Comments to the Author” section, enter your conflict of interest statement in the “Confidential to Editor” section, and submit your "Accept" recommendation.

Reviewer #4: (No Response)

Reviewer #7: All comments have been addressed

**********

2. Does this manuscript meet PLOS Global Public Health’s publication criteria? Is the manuscript technically sound, and do the data support the conclusions? The manuscript must describe methodologically and ethically rigorous research with conclusions that are appropriately drawn based on the data presented.

Reviewer #4: Yes

Reviewer #7: Yes

**********

3. Has the statistical analysis been performed appropriately and rigorously?

Reviewer #4: Yes

Reviewer #7: Yes

**********

4. Have the authors made all data underlying the findings in their manuscript fully available (please refer to the Data Availability Statement at the start of the manuscript PDF file)?

The PLOS Data policy requires authors to make all data underlying the findings described in their manuscript fully available without restriction, with rare exception. The data should be provided as part of the manuscript or its supporting information, or deposited to a public repository. For example, in addition to summary statistics, the data points behind means, medians and variance measures should be available. If there are restrictions on publicly sharing data—e.g. participant privacy or use of data from a third party—those must be specified.

Reviewer #4: Yes

Reviewer #7: Yes

**********

5. Is the manuscript presented in an intelligible fashion and written in standard English?

PLOS Global Public Health does not copyedit accepted manuscripts, so the language in submitted articles must be clear, correct, and unambiguous. Any typographical or grammatical errors should be corrected at revision, so please note any specific errors here.

Reviewer #4: Yes

Reviewer #7: Yes

**********

6. Review Comments to the Author

Please use the space provided to explain your answers to the questions above. You may also include additional comments for the author, including concerns about dual publication, research ethics, or publication ethics. (Please upload your review as an attachment if it exceeds 20,000 characters)

Reviewer #4: Title : The survival penalty : age-based inequities in hypertension and diabetes managementin African health systems (1990–2022) – a secondary quantitative analysis

Version : 22 May 2026

Reviewer : Paulin Nkolamoyo Musungula

Reviewer's report : The authors have overall responded in a thorough and satisfactory manner to all methodological comments. Without questioning the overall quality of the work, I remain nevertheless concerned about several points related to interpretation and wording, which would benefit from adjustment in order to ensure strict alignment between the data and the conclusions.

1. The following formulation remains problematic: “which likely leads to an underestimation of the true survival penalty (Line 370)”. It implicitly assumes the existence of a mechanism (“survival penalty”) that is not demonstrated by the study. A more neutral formulation would be preferable, for example: “may underestimate age-related differences in hypertension indicators”, in order to remain strictly descriptive and consistent with an ecological study design.

2. The manuscript does not explicitly discuss survivorship bias (healthy survivor effect), which is nevertheless essential for the interpretation of older age groups (75–79 years and ≥85 years). An appropriate correction would be to add an explicit limitation such as: “observed age gradients may partly reflect survivorship bias, as older age groups represent a selected subset of healthier survivors”.

3. Several statements remain overly interpretative, in particular: “implicit rationing (Line 381)”, “systems functioning in ways that are implicitly age-stratified (Line 392)”, and “care intensity does not consistently correlate with clinical need (Line 394)”. These formulations imply unmeasured mechanisms. A more rigorous version would be to rephrase in terms of descriptive associations, for example: “observed age-related differences in care cascade indicators suggest heterogeneity across age groups, without allowing inference on underlying mechanisms or on the appropriateness of care intensity”.

Reviewer #7: The authors have addressed all previous reviewers' comments adequately.

**********

7. PLOS authors have the option to publish the peer review history of their article (what does this mean?). If published, this will include your full peer review and any attached files.

**Do you want your identity to be public for this peer review?**  If you choose “no”, your identity will remain anonymous but your review may still be made public.

For information about this choice, including consent withdrawal, please see our Privacy Policy.

Reviewer #4: No

Reviewer #7: No

**********

[NOTE: If reviewer comments were submitted as an attachment file, they will be attached to this email and accessible via the submission site. Please log into your account, locate the manuscript record, and check for the action link "View Attachments". If this link does not appear, there are no attachment files.]

Figure Resubmissions:

While revising your submission, we strongly recommend that you use PLOS’s NAAS tool (https://ngplosjournals.pagemajik.ai/artanalysis) to test your figure files. NAAS can convert your figure files to the TIFF file type and meet basic requirements (such as print size, resolution), or provide you with a report on issues that do not meet our requirements and that NAAS cannot fix.

After uploading your figures to PLOS’s NAAS tool - https://ngplosjournals.pagemajik.ai/artanalysis, NAAS will process the files provided and display the results in the "Uploaded Files" section of the page as the processing is complete. If the uploaded figures meet our requirements (or NAAS is able to fix the files to meet our requirements), the figure will be marked as "fixed" above. If NAAS is unable to fix the files, a red "failed" label will appear above. When NAAS has confirmed that the figure files meet our requirements, please download the file via the download option, and include these NAAS processed figure files when submitting your revised manuscript.

# Author comment: Author response to Decision Letter 2

**DOI:** 10.1371/journal.pgph.0006306.r004

19 Jun 2026

Attachment Submitted filename: Response to Reviewers PLOS - R2.docx

# Editor report: Decision Letter 2

**DOI:** 10.1371/journal.pgph.0006306.r005

**Authors:** Priyanka Roy

30 Jun 2026

The survival penalty: age-based inequities in hypertension and diabetes management in African health systems (1990–2022) – a secondary quantitative analysis.

PGPH-D-26-00316R2

Dear Dr Basil,

We are pleased to inform you that your manuscript 'The survival penalty: age-based inequities in hypertension and diabetes management in African health systems (1990–2022) – a secondary quantitative analysis.' has been provisionally accepted for publication in PLOS Global Public Health.

Before your manuscript can be formally accepted you will need to complete some formatting changes, which you will receive in a follow up email. A member of our team will be in touch with a set of requests.

Please note that your manuscript will not be scheduled for publication until you have made the required changes, so a swift response is appreciated.

IMPORTANT: The editorial review process is now complete. PLOS will only permit corrections to spelling, formatting or significant scientific errors from this point onwards. Requests for major changes, or any which affect the scientific understanding of your work, will cause delays to the publication date of your manuscript.

If your institution or institutions have a press office, please notify them about your upcoming paper to help maximize its impact. If they'll be preparing press materials, please inform our press team as soon as possible -- no later than 48 hours after receiving the formal acceptance. Your manuscript will remain under strict press embargo until 2 pm Eastern Time on the date of publication. For more information, please contact globalpubhealth@plos.org.

Thank you again for supporting Open Access publishing; we are looking forward to publishing your work in PLOS Global Public Health.

Best regards,

Priyanka Roy, MD, MPH, PhD

Academic Editor

PLOS Global Public Health

***********************************************************

Reviewer Comments (if any, and for reference):
